# Clinical application of the tibial extension stem in primary TKA for knee varus deformity combined with tibial bone defect

**DOI:** 10.3389/fsurg.2025.1558338

**Published:** 2025-03-18

**Authors:** Fei Yuan, Yankun Li, Xiaogang Shen, Xuepeng Zhu, Li Sun, Youliang Ren, Tao Guo, Bo Li

**Affiliations:** ^1^ Department of Orthopaedic Surgery, Guizhou Provincial People’s Hospital, Guiyang, China; ^2^ Department of Zunyi Medical University, Zunyi, China

**Keywords:** total knee arthroplasty, knee varus deformity, tibial bone defect, the tibial extension stem, surgery

## Abstract

**Objective:**

To observe the clinical efficacy of prophylactic use of tibial extension stem in primary Total Knee Arthroplasty (TKA) in patients with severe knee varus deformity and tibial plateau bone defect, and its effect on reducing the rate of tibial prosthesis aseptic loosening.

**Methods:**

A total of 398 patients who underwent primary TKA in our hospital from August 2019 to June 2021 were collected. According to the strict inclusion/exclusion criteria, 55 patients with knee varus deformity (Hip-knee-ankle Angle, HKA ≤ 160°) and tibial bone defect were finally included. The tibial extension stem was used in 22 patients and standard tibial prosthesis was used in 33 patients. The general data, intraoperative parameters, preoperative and postoperative imaging parameters and knee function scores (KSS) were analyzed. The incidence of tibial prosthesis aseptic loosening, KSS score, radiological evaluation of the prosthesis and bone cement screws, and postoperative general complications were dynamically followed up and evaluated.

**Results:**

All 55 patients completed long-term follow-up, with an average follow-up time of 46.1 ± 4.2 months. There was no significant difference in HKA Angle between the two groups before and after operation (*p* > 0.05). At least 36 months follow-up, The final Society Radiographic Evaluation System (KSRES) scores were significantly different in the range of 4–10 mm (*p* < 0.05), but no screw loosening, sinking, osteolysis, bone cement fracture and serious postoperative complications occurred in all patients. There was no significant difference in KSS scores between the two groups during the follow-up period (*p* > 0.05). At the end of follow-up, there was no aseptic loosening of tibial prosthesis and serious postoperative complications in both groups.

**Conclusions:**

For patients with severe knee varus deformity and tibial plateau bone defect, the use of tibial extension stem in primary TKA may have a protective effect on the survival rate of prosthesis.

## Introduction

Knee Osteoarthritis (OA), a common degenerative joint disease, is also one of the main causes of disability and chronic pain in patient's (1, 2). TKA is currently an effective and highly successful surgical method for treating primary Knee OA (3). In the past few decades, the number of total knee joint revisions after initial knee arthroplasty has rapidly increased worldwide. Although the clinical application of TKA has become quite mature in recent years, with the increase in the number of knee joint replacements, the incidence of postoperative complications has also increased, with the most common being aseptic loosening and infection. Research has confirmed that patients with severe inversion of the knee joint have a higher rate of loosening and revision after TKA surgery than do those with milder deformities (4–8). Previous studies have shown that improvements in tibial implants may effectively address this issue. The tibial extension stem are often used in complex initial and revision TKA, with the benefit of reducing tibial detachment and lateral shear stress (9, 10). In addition, the advantages of using a short the tibial extension stem include effectively dispersing stress at the prosthesis-bone interface, maintaining the stability of the prosthesis, reducing the probability of prosthesis loosening (11, 12), reducing micromovement, strengthening fixation, and potentially reducing the risk of pain (9, 13, 14).

Recently, an increasing number of researchers have been interested in the prophylactic use of the tibial extension stem in TKA surgery, as prophylactic use of the tibial extension stem has become a common surgical technique. This technology can effectively solve some problems that arise after TKA surgery and achieve better surgical results. However, there are currently few reports on whether prophylactic use of the tibial extension stem in TKA is more advantageous for reducing postoperative revision rates in patients with severe knee joint varus deformity combined with tibial plateau bone defects. Therefore, the purpose of this study was to observe whether prophylactic use of the tibial extension stem can reduce the incidence of early aseptic loosening of prostheses in patients with severe knee varus deformity combined with tibial plateau bone defects during initial TKA.

## Materials and methods

**Figure 1:**
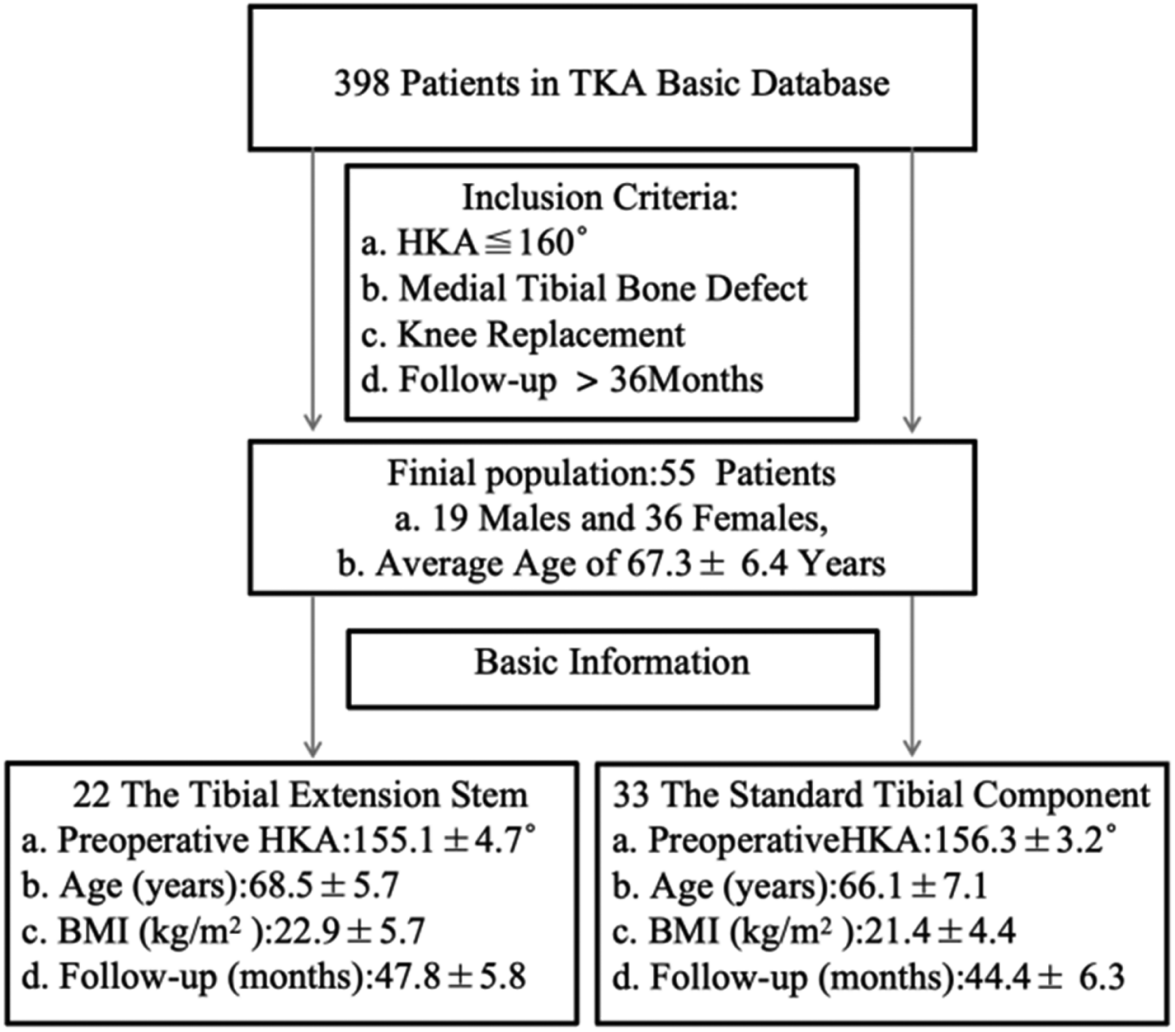
Flowchart of the study.

The data for this retrospective study were based on a prospective collection of 398 TKA surgeries performed by the same team of joint surgeons at our institution from August 2019 to June 2021, with all surgical options selected for total knee arthroplasty. The inclusion criteria for this study included the following: (1) Knee joint HKA ≤ 160°; (2) Combined with tibial plateau bone defect (tibial plateau defect depth ≤10 mm, unilateral tibial plateau defect area <50%); (3) Perform primary TKA; (4) The follow-up time was at least 36 months. After screening, A total of 55 patients were enrolled, including 19 males and 36 females. All the patients were divided into two cohorts for observation, including 22 cases with 10mm × 40 mm the tibial extension stem and 33 cases with standard tibial prosthesis (Figure 1). For patients with tibial plateau bone defect depth <5 mm, autologous bone graft was used to fill the defect, and for patients with defect depth 5–10 mm, bone cement screw was used to support the defect.

**Figure 2:**
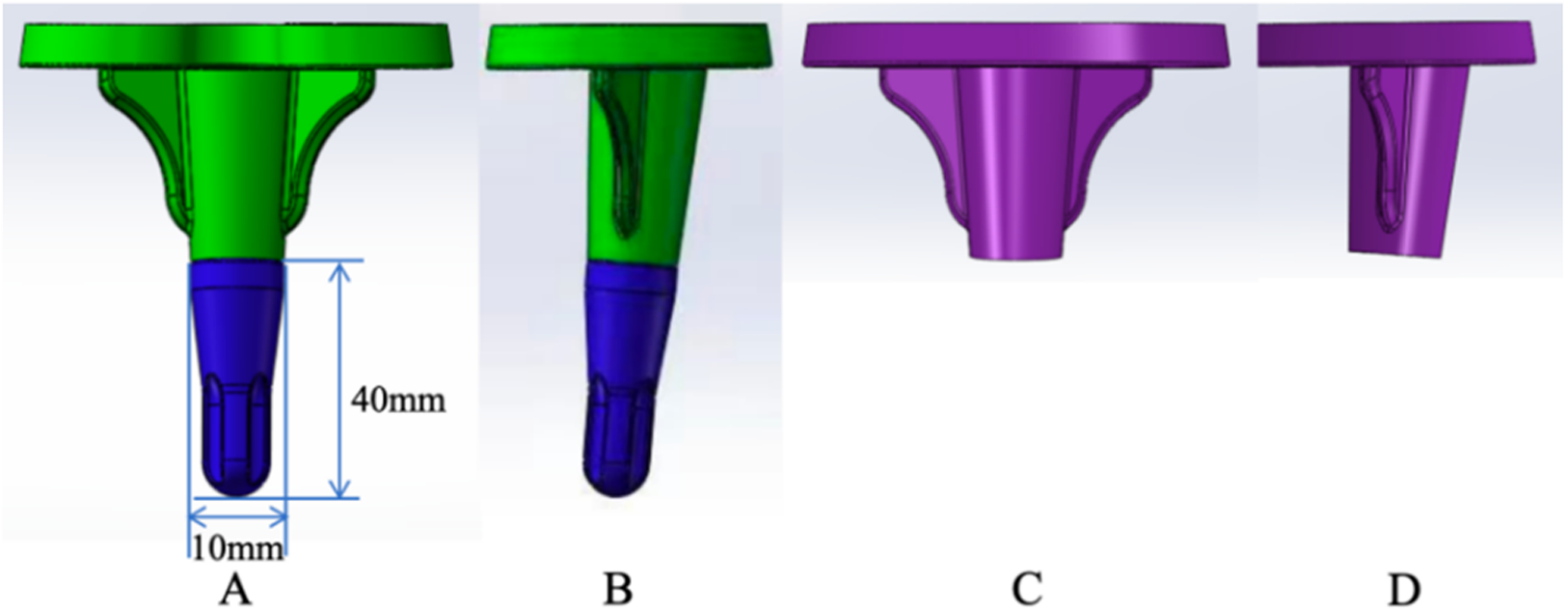
The tibial extension stem and standard tibial prosthesis implant; **(A**,**B)** the tibial extension stem tibial prosthesis anteroposterior and lateral position, **(C,D)** standard tibial prosthesis anteroposterior and lateral position.

The artificial joint implants used in both queues were sourced from Chunli Zhengda Medical Equipment Co., Ltd. (Beijing, China) (Figure 2). The average follow-up time after surgery was 46.1 ± 4.2 months. General information such as sex, age, and BMI was collected from all patients before surgery, and imaging data such as full-length films of both lower limbs and anterior posterior (AP) and lateral x-ray translucency lines were regularly obtained after surgery. Based on these findings, the hip knee ankle angle (HKA) and radiolucency line (RLL) were measured. Additionally, the American Knee Society Scale (KSS) is used for functional assessment.

### Operative technique

A medial parapatellar approach was used in both groups, and the procedure was performed in the supine position with a balloon tourniquet applied at the root of the thigh on the affected side and pressure set at 280mmHg. After removing the distal femur and proximal tibia, the soft tissue around the knee joint was released to achieve the balance of tension between the medial and lateral soft tissue. Then the femoral prosthesis, tibial prosthesis and shim model were implanted (15). The lower limb alignment was normal, and the range of motion was from extension (0°) to flexion (120°). Autogenous bone or cement screws were implanted in the bone defect of tibial plateau, and the femoral and tibial prostheses and polyethylene pads were inserted to reduce the joint. After the bone cement was solidified, the stability of flexion and extension of the joint was good, and the position of the artificial joint prosthesis was confirmed by intraoperative x-ray film, and the operation was finished.

### Follow-up and evaluation of tibial loosening

**Figure 3:**
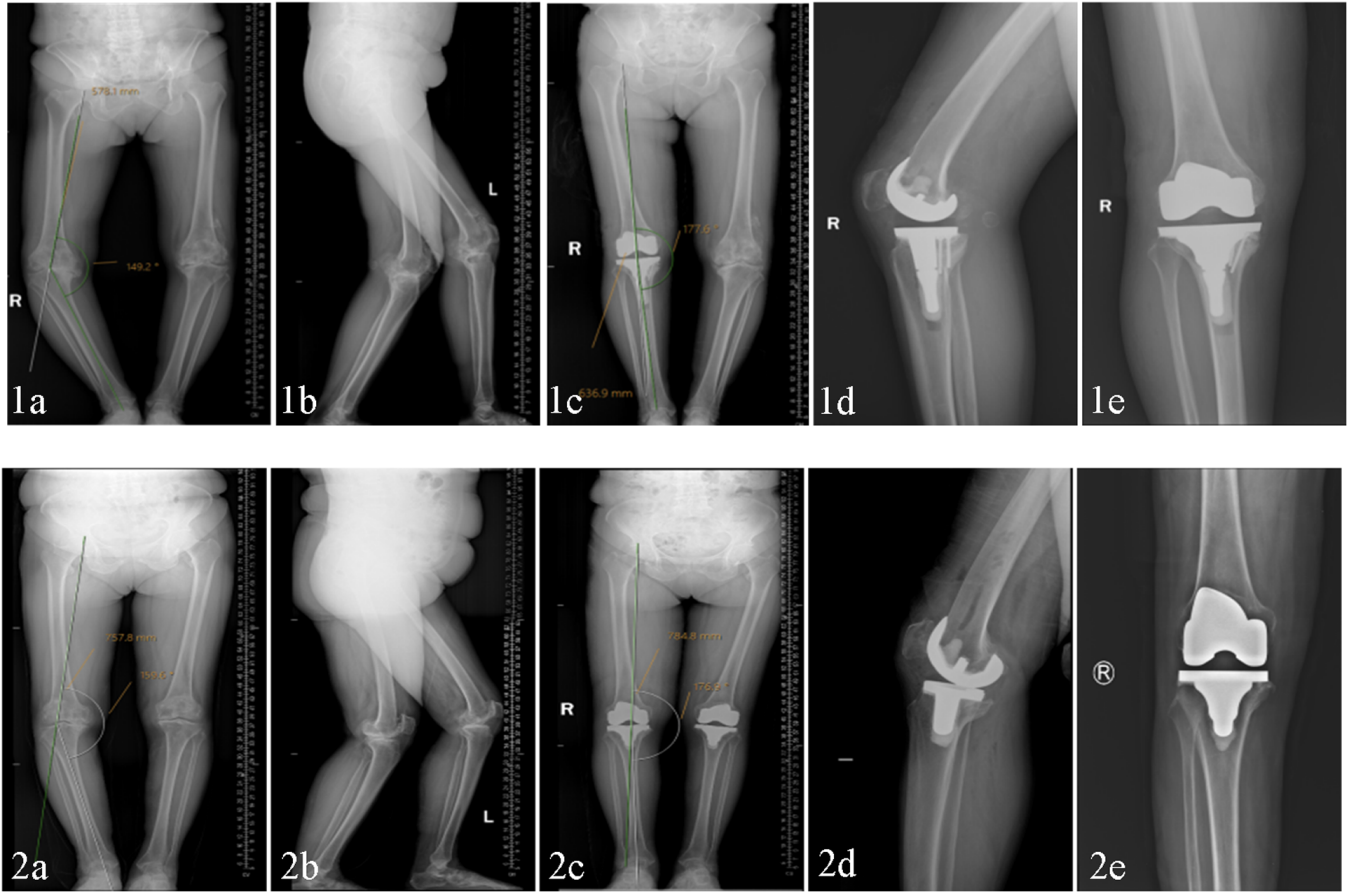
The tibial extension stem group **(1)** and standard tibial prosthesis group **(2)** were compared in terms of preoperative and postoperative DR and HKA angles; **(a**,**b)** preoperative full-length anteroposterior lateral (DR) and preoperative HKA angle of both lower limbs. **(c)** Postoperative 36-month HKA angle. **(d**,**e)** Postoperative 36-month knee joint AP and lateral positions.

Radiological data were obtained before surgery and 36 months after surgery, including full-length radiographs of both lower limbs, anterior-posterior (AP) and lateral radiographs of the knee joint, and radiolucosal lines and HKA angles were measured (Figure 3). Follow-up and evaluation were performed by two specialists who were not involved in this operation to evaluate the results by radiographic measurements and the KSS scores at 36 months after surgery (16).

**Figure 4:**
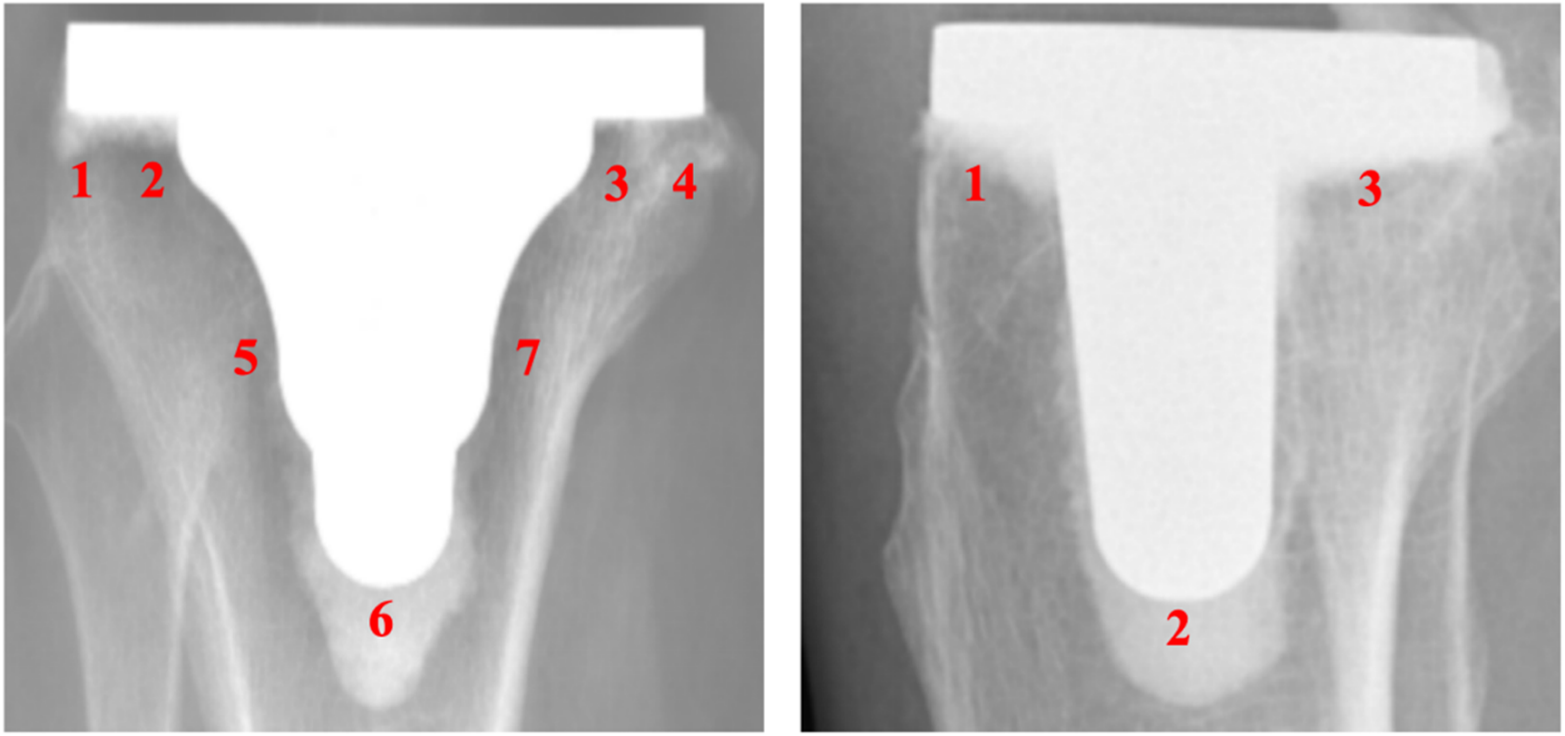
Knee joint AP and lateral x-ray images, divided according to the American knee association TKA postoperative x-ray evaluation system (KSRES score).

All patients had Anterior Posterior (AP) and lateral x-ray images of the knee joint taken during follow-up at least 36 months after surgery, and the preoperative and postoperative HKA angles were measured using our institution's image storage system (Figure 3). Radiographic transparency was also measured by two professional orthopedic physicians who did not participate in the surgery (Figure 4). According to Meneghini et al. (17) and Chalmers et al. (18), the KSRES scoring system for total knee arthroplasty in the United States is allocated based on the cumulative number of radiolucent lines on knee AP and lateral x-rays. The scoring of AP and lateral x-rays was determined by measuring the width of the radial translucency line in millimeters for each area of the AP and lateral x-rays. The sum of the widths of each area was the total score. The KSRES score was less than or equal to 4 millimeters, 4–10 mm, or greater than or equal to 10 mm. To evaluate whether there was aseptic loosening of the tibial prosthesis, the score was evaluated as follows: 4 points or lower, indicating no progression or possible insignificance; a score of 4–10 indicated that looseness did not occur but may have had a tendency to occur. Regular reexamination and close monitoring of progress was conducted; a score of 10 or higher indicated possible or imminent loosening regardless of symptoms. A preliminary diagnosis of implant loosening was made by combining clinical and imaging techniques. Patients suspected of implant loosening underwent blood tests (C-reactive protein concentration, white blood cell count, and erythrocyte sedimentation rate), CT scans, and other techniques to confirm the occurrence of aseptic loosening.

### Statistical analysis

The measured parameters between the two groups were expressed as mean ± standard deviation (x ± s). The preoperative and postoperative HKA, KSS scores and implant loosening were analyzed by one-way ANOVA and t test, respectively. *P* < 0.05 was considered statistically significant, and all statistical analyses were performed using the Statistical Product and Service Solutions (SPSS) version 23.0 (SPSS Inc., Chicago, Illinois, USA).

## Results

### General information

There were no differences in age, sex, BMI, follow-up time, or surgical time between the tibial extension stem group and the standard tibial prosthesis group based on comparisons of the preoperative general information (Table 1).

**Table 1:** Pre-operative population characteristics in the two study groups.

Type	Total	Extension stem	Standard stem	*p* value
Patient	55	22	33	ns
Male	19	9	10	ns
Female	36	13	23	ns
Age (years) (mean ± SD) [Min; Max]		68.5 ± 5.7 [52; 78]	66.1 ± 7.1 [52; 79]	ns
BMI (kg/m^2^) (mean ± SD) [Min; Max]		22.9 ± 5.7 [18.1; 32.3]	21.4 ± 4.4 [17.5; 34.6]	ns
Follow-up (months) (mean ± SD) [Min; Max]		47.8 ± 5.8 [36; 58]	44.4 ± 6.3 [36; 58]	ns

SD, standard deviation; Min, minimum; Max, maximum; ns, nonsignifcant.

### Surgical outcomes

The preoperative HKA Angle of the tibial extension stem group was smaller than that of the standard tibial prosthesis group according to the evaluation of 2 orthopedic surgeons who were not involved in the operation (155.1° ± 4.7° vs. 156.3° ± 3.2°, *P* > 0.05). However, no statistically significant difference was found between the tibial extension stem group and the standard tibial prosthesis group in terms of postoperative HKA. (178.3° ± 3.9° vs. 177.9° ± 4.1°, *P* > 0.05) (Table 2).

**Table 2:** Post-operative results.

Indicators	Extension stem	Standard stem	*p* value
KSS knee score (mean ± SD) [Min; Max]	87.5 ± 3.7 [78; 93]	87.0 ± 4.6 [77; 96]	ns
KSS function score (mean ± SD) [Min; Max]	78.0 ± 5.4 [68; 88]	77.1 ± 3.9 [70; 87]	ns
Preoperative HKA (°) (mean ± SD) [Min; Max]	155.1 ± 4.7 [151.5; 159.8]	156.3 ± 3.2 [154.1; 159.9]	ns
Postoperative HKA (°) (mean ± SD) [Min; Max]	178.3 ± 3.9 [175.6; 179.5]	177.9 ± 4.1 [168.5; 179.6]	ns
Tourniquet (min) (mean ± SD) [Min; Max]	68.2 ± 16.7 [55; 118]	70.6 ± 17.8 [61; 125]	ns

SD, standard deviation; Min, minimum; Max, maximum; ns, nonsignifcant.

### Postoperative complications

The mean follow-up time was 46.1 ± 4.2 months. There were no serious postoperative complications of tibial prosthesis aseptic loosening, screw loosening, sinking, osteolysis, bone cement fracture and infection in the the tibial extension stem group and the standard tibial prosthesis group.

### KSS and KSS functional scores

The KSS (87.5 ± 3.7 points vs. 87.0 ± 4.6 points, *P* > 0.05) and KSS (78.0 ± 5.4 points vs. 77.1 ± 3.9 points, *P* > 0.05) indicated that there was no significant difference between the short tibial extension rod group and the standard tibial prosthesis group (Table 2).

### Radiological measurements

Radiological parameters were measured at least 36 months after surgery in all patients. There was no significant difference in the occurrence of aseptic loosening between the tibial extension stem group and standard tibial prosthesis group in AP and lateral positions (*P* > 0.05).There was no significant difference in KSRES score between the tibial extension stem group and the standard tibial prosthesis group in the range of ≤4 mm and ≥10 mm. However, in the standard tibial prosthesis group, there were 6 patients (18%) with KSRES score between 4 and 10 mm. Pearson's chi-square test showed that there was a significant difference between the two groups (*p* < 0.05). The results showed that the risk of aseptic loosening in the standard tibial prosthesis group was higher than that in the short tibial prosthesis group, and regular review and close attention should be paid to its progress (Table 3).

**Table 3:** Radiations were measured on positive and lateral radiographs 36 months after surgery.

KSRES rating	Extension stem	Standard stem	*p* value
≤4 mm	22 (100%)	27 (82%)	ns
4–10 mm	0	6 (18%)	*P* = 0.034^a^
10 mm	0	0	ns
Total	22	33	

ns, nonsignifcant.

^a^ Pearson Chi-square Test.

## Discussion

In this clinical study, 55 patients with severe knee varus deformity combined with tibial bone defect were divided into two cohorts for observation. Through at least 36 months of follow-up, radiographic data showed that there were no serious postoperative complications of tibial prosthesis aseptic loosening, screw loosening, subsidence, osteolysis, bone cement fracture and infection in the tibial extension stem group and the standard tibial prosthesis group.All radiolucent line measurement (RLL) KSRES scores were less than 10 in both groups. No direct evidence of imminent or possible failure was found. Although there was no significant difference in the survival rate of prosthesis between the two groups in this study, the KSRES score in the 4–10 mm range was 6 cases (18%) in the standard tibial prosthesis group, and 0 case in the tibial extension stem group, there was a significant difference between the two groups (*p* < 0.05). The results indicate that these 6 patients may have a tendency and progression of loosening. The risk of aseptic loosening in the standard tibial component group is higher than that in the tibial extension stem group. The risk of implant stability reduction is higher, suggesting that the prophylactic use of the tibial extension stem in primary TKA may protect the survival rate of prosthesis in patients with severe knee varus deformity and tibial bone defect.

The inclusion of a the tibial extension stem at the lower end of the tibial prosthesis platform is a common practice aimed at enhancing the stability of the tibial plateau prosthesis and distributing the body's weight evenly. Previous studies have shown that prophylactic use of the tibial extension stem can reduce the risk of postoperative complications and improve the treatment outcome of patients. the tibial extension stem can improve the clinical efficacy of patients and improve the survival rate of prostheses by increasing the contact area of the tibial implant-bone interface, thereby improving the stability of tibial prosthesis and reducing a certain amount of tibial pressure. At the same time, Glenn et al. (14) and Hegde et al. (19) showed that prophylactic use of a short tibial extension resulted in a lower incidence of aseptic loosening in patients with preoperative genu varum deformity, because the short tibial extension could reduce compression stress by 136% and shear stress at the bone-cement interface by 92%. Moreover, a study conducted by Fournier (20) et al. demonstrated that the proactive adoption of the tibial extension stem during primary total knee arthroplasty may effectively mitigate the aforementioned issues. In this rigorously conducted study comprising 180 patients, the rate of tibial implant loosening was 3% in the cohort lacking the tibial extension stem, while the corresponding rate was 0% in the cohort equipped with such stem. It is widely acknowledged that obesity is linked to heightened postoperative complications and diminished prognosis scores subsequent to total knee arthroplasty (TKA). In a study conducted by Steere et al. with an average follow-up duration of 34 months, neither group experienced any instances of aseptic loosening failure, irrespective of the use of prophylactic the tibial extension stem (21). Furthermore, quantitative analysis revealed no significant difference in the percentage of RLL between the two groups. Research conducted by Abdel MP (22) et al. revealed that the use of abbreviated extension stem in TKA procedures for obese individuals can effectively diminish the occurrence of tibial loosening, as observed during a minimum two-year postoperative monitoring period.

The novelty of this study lies in the fact that it is the first comparison between patients with preoperative HKA ≤ 160° and combined tibial plateau bone defects in TKA surgery and whether prophylactic use of the tibial extension stem is used. Preventive use of the tibial extension stem can effectively repair tibial varus deformities in patients with tibial bone defects, restore good lower limb force lines, improve gait and pain symptoms, restore motor function, and improve quality of life. This brings good news to patients with primary knee osteoarthritis with severe genu varum deformity and tibial plateau bone defect, and has very important guiding significance for their clinical diagnosis and treatment. Moreover, during postoperative follow-up observation, if the KSRES score is found to be within the 4–10 mm range, patients should receive more regular follow-up and close monitoring of their progression. At the same time, patients should be educated regarding safety measures, such as reducing high-intensity activities and avoiding falls and collisions. However, there are still many shortcomings in this study, such as the small sample size, short follow-up period, and the possibility of some patients missing serious complications and experiencing aseptic loosening due to being lost to follow-up. It is necessary to include more patients and extend the follow-up time in future studies to further verify the long-term efficacy of these regimens. In summary, the preventive use of the tibial extension stem has been widely used in TKA surgery, but its effectiveness still needs further research. At present, the use of a the tibial extension stem may be an effective treatment for patients with severe knee varus deformity combined with tibial bone defects, but sufficient preoperative planning and risk assessment should be conducted before surgery to ensure its safety and effectiveness, and correct surgical decisions should be made. Personalized treatment should be conducted with an approach clinically based on the specific circumstances.

## Conclusion

For patients with severe knee varus deformity and tibial plateau bone defect, the use of tibial extension stem in primary TKA may have a protective effect on the survival rate of prosthesis.

## Data Availability

The datasets presented in this study can be found in online repositories. The names of the repository/repositories and accession number(s) can be found in the article/Supplementary Material.
